# Supplementary material for: Structural insights into the modulation of coronavirus spike tilting and infectivity by hinge glycans
Source: Nat Commun. 2023 Nov 7;14:7175. doi: 10.1038/s41467-023-42836-9 (PMC10630519; doi:10.1038/s41467-023-42836-9)
Supplement: Supplementary file 1 — Supplementary Information [file 41467_2023_42836_MOESM1_ESM.pdf]

Structural Insights Into the Modulation of Coronavirus Spike Tilting and Infectivity by Hinge Glycans

David Chmielewski[1†], Eric A. Wilson[2,†], Greg Pintilie[3], Peng Zhao[4], Muyuan Chen[5], Michael F. Schmid[5], Graham Simmons[6,7], Lance Wells[4], Jing Jin[6,7*], Abhishek Singharoy[2*], Wah Chiu[1,3,5*]

[1]Biophysics Graduate Program, Stanford University, Stanford, CA 94305, USA

[2]School of Molecular Sciences, Biodesign Institute, Arizona State University, Tempe, AZ USA

[3]Department of Bioengineering, and of Microbiology and Immunology, Stanford University, Stanford, CA 94305, USA

[4]Complex Carbohydrate Research Center, University of Georgia, Athens, GA 30602, USA

[5]Division of CryoEM and Bioimaging, SSRL, SLAC National Accelerator Laboratory, Stanford University, Menlo Park, CA 94025, USA

[6]Vitalant Research Institute, San Francisco, CA, 94118, USA

[7]Department of Laboratory Medicine, University of California, San Francisco, San Francisco, CA, 94143, USA

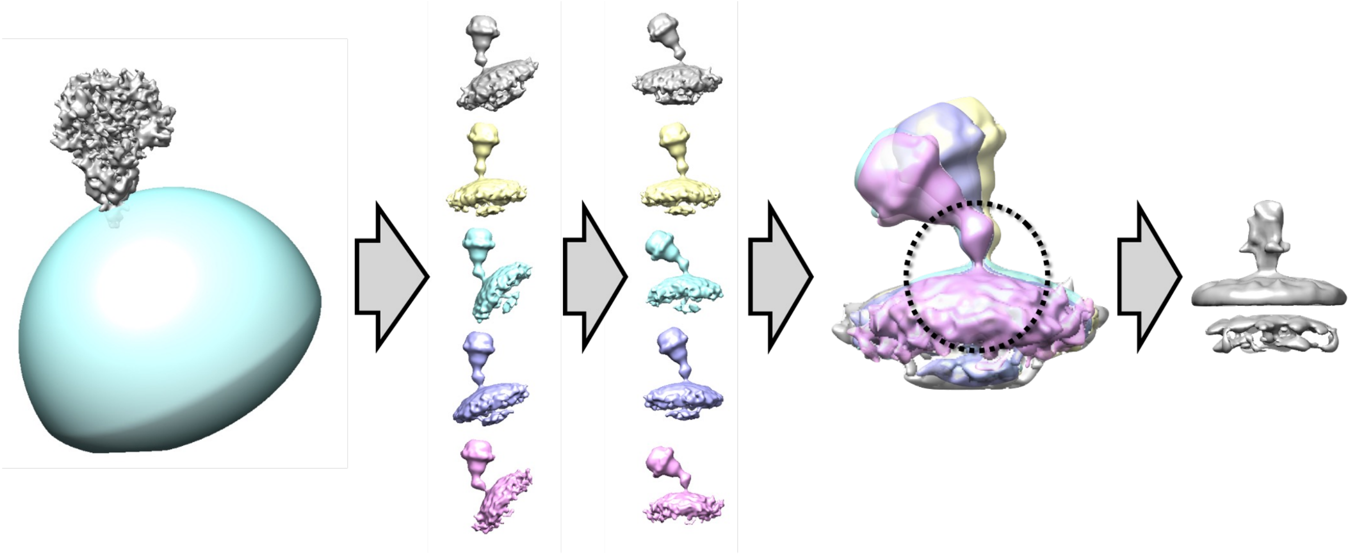

Supplementary Figure 1. Schematic presentation of subtomogram analysis of the spike and stalk on HCoV-NL63 virion (related to Figure 1)

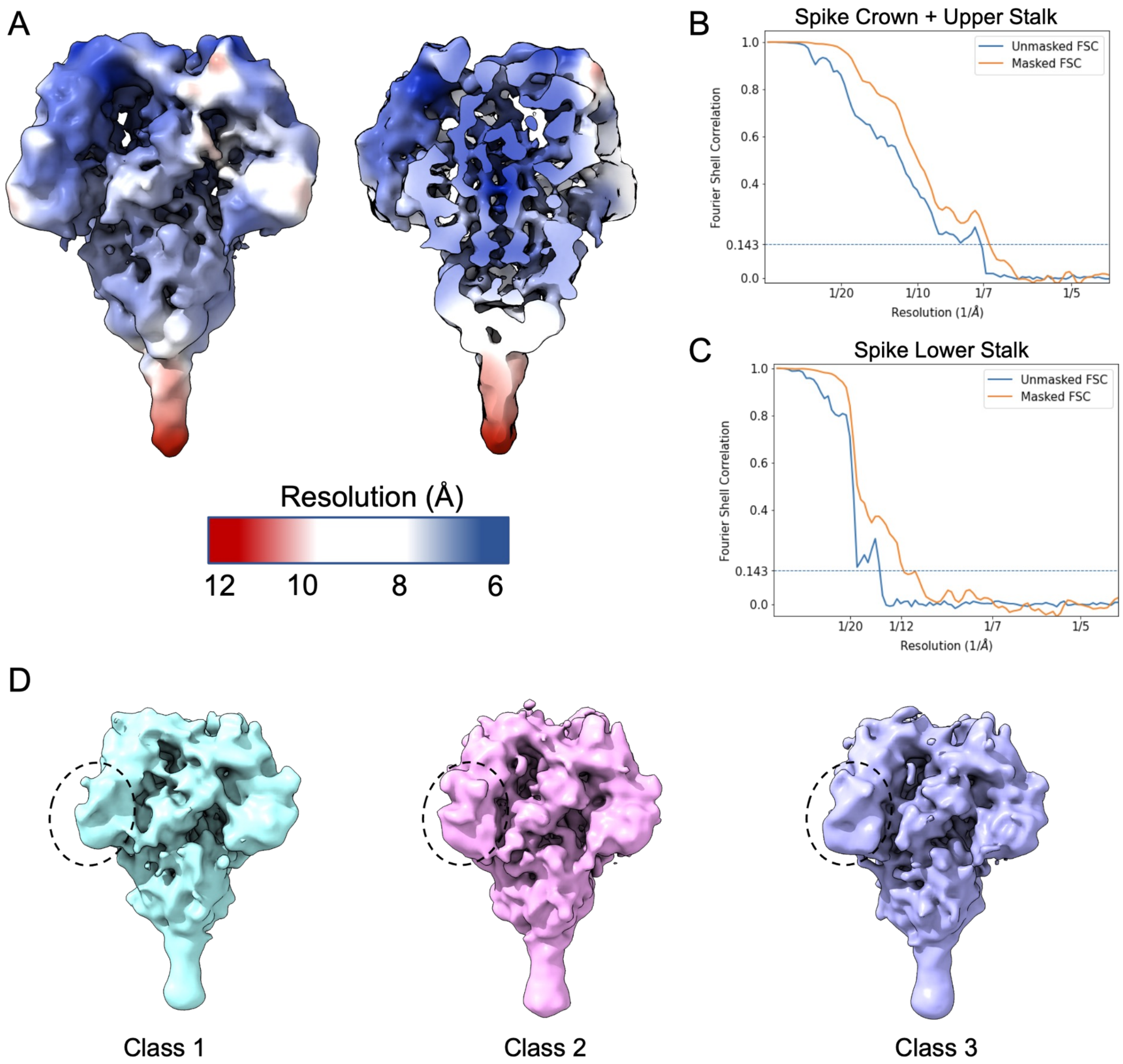

Supplementary Figure 2. Resolution assessment and classification analysis of subtomogram average structures (related to Figure 1). (A) Subtomogram average of the HCoV-NL63 spike crown and upper stalk region (left) with half-cut representation (right) colored by local resolution as determined by ResMap (PMID: 24213166). (B) Masked and unmasked FSC plot of S crown and upper stalk (C) A FSC plot of S lower stalk as measured by gold-standard FSC (0.143 criterion). (D) 3D classification of spike crowns and upper stalks focused on domain 0 region (dashed black circle) reveals lack of alternate domain 0 conformations.

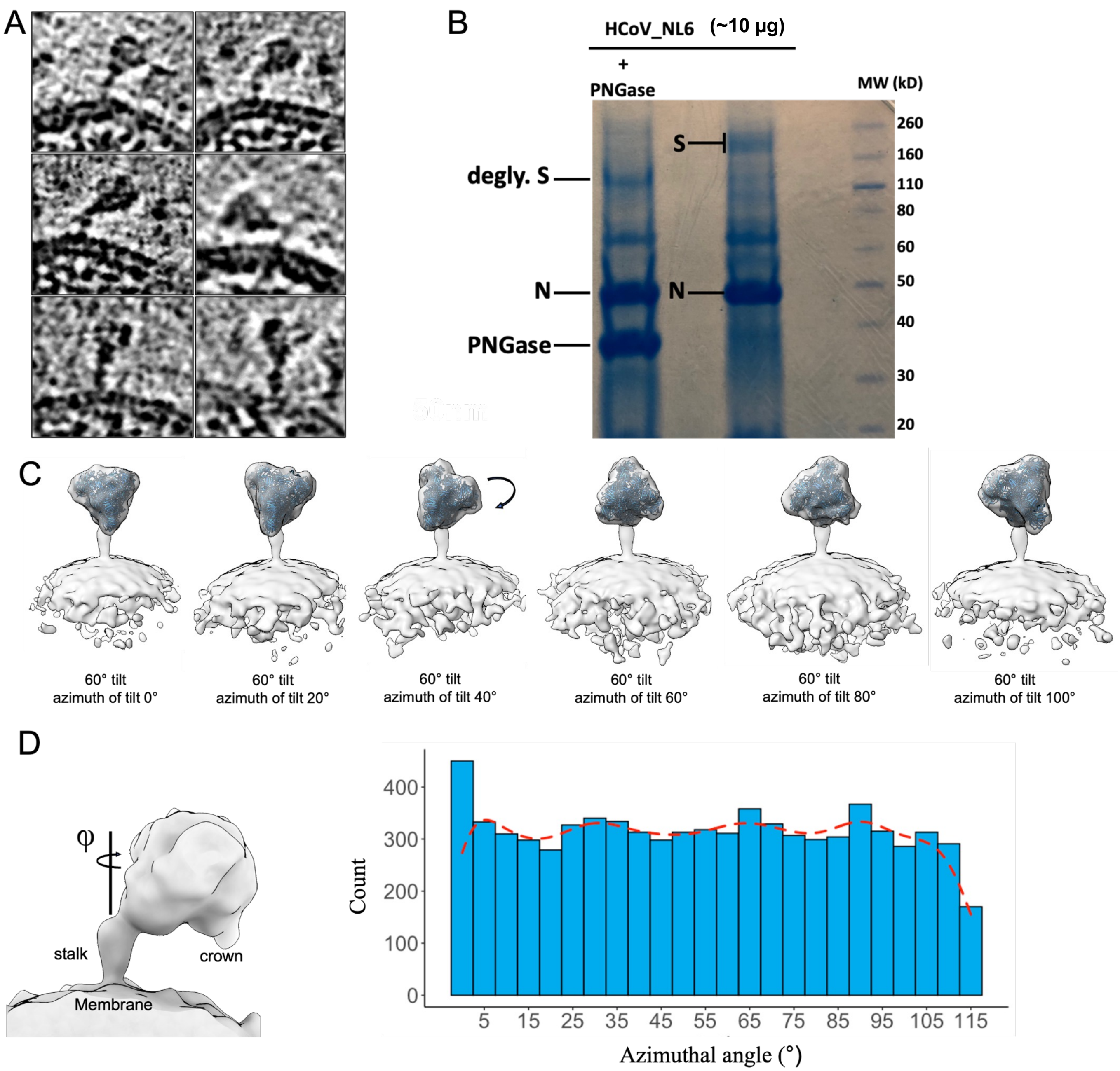

Supplementary Figure 3. Spike dynamics on intact virions (related to Figure 1). (A) Tomographic slice images of spikes on the membrane of intact particles. (B) Purified HCoV-NL63 and PNGase digested virus were subjected to SDS-PAGE gel analysis followed by Coomassie-staining. One representative result from > 3 reproducible experiments is shown. (C) Six subtomogram class averages of spikes at different azimuthal direction determined for spikes at 60 degrees tilt angle. (D) Distributions of azimuth of tilt angle of crown relative to stalk for each spike, determined from the refined subvolume orientations of crown and stalk regions. Source data are provided as a Source Data file.

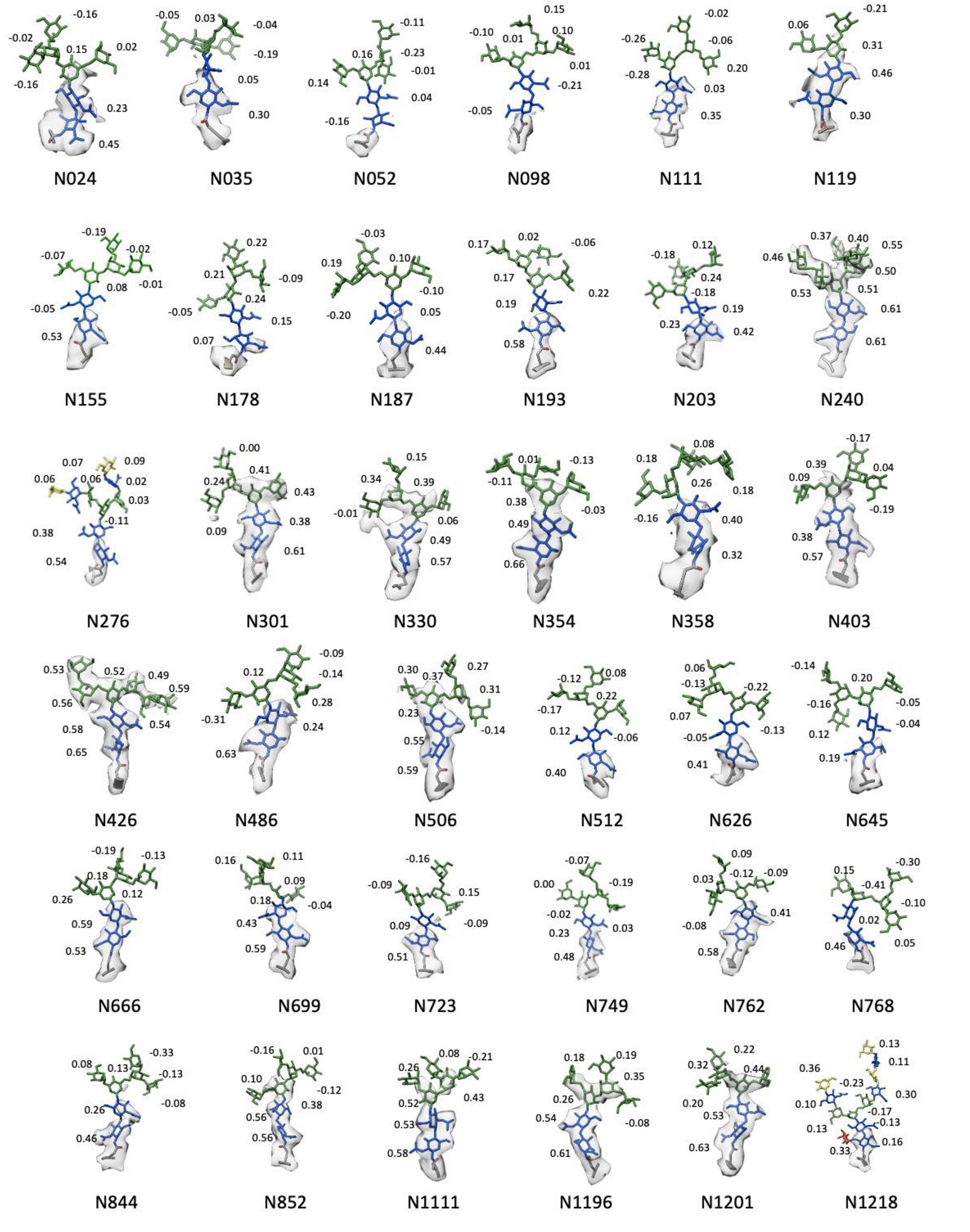

Supplementary Figure 4. Asn amino acid residues in the crown and attached glycans with extracted densities (EMD-29395) and Q-scores from the single particle cryoEM structure (PDB:7KIP) (related to Figure 2).

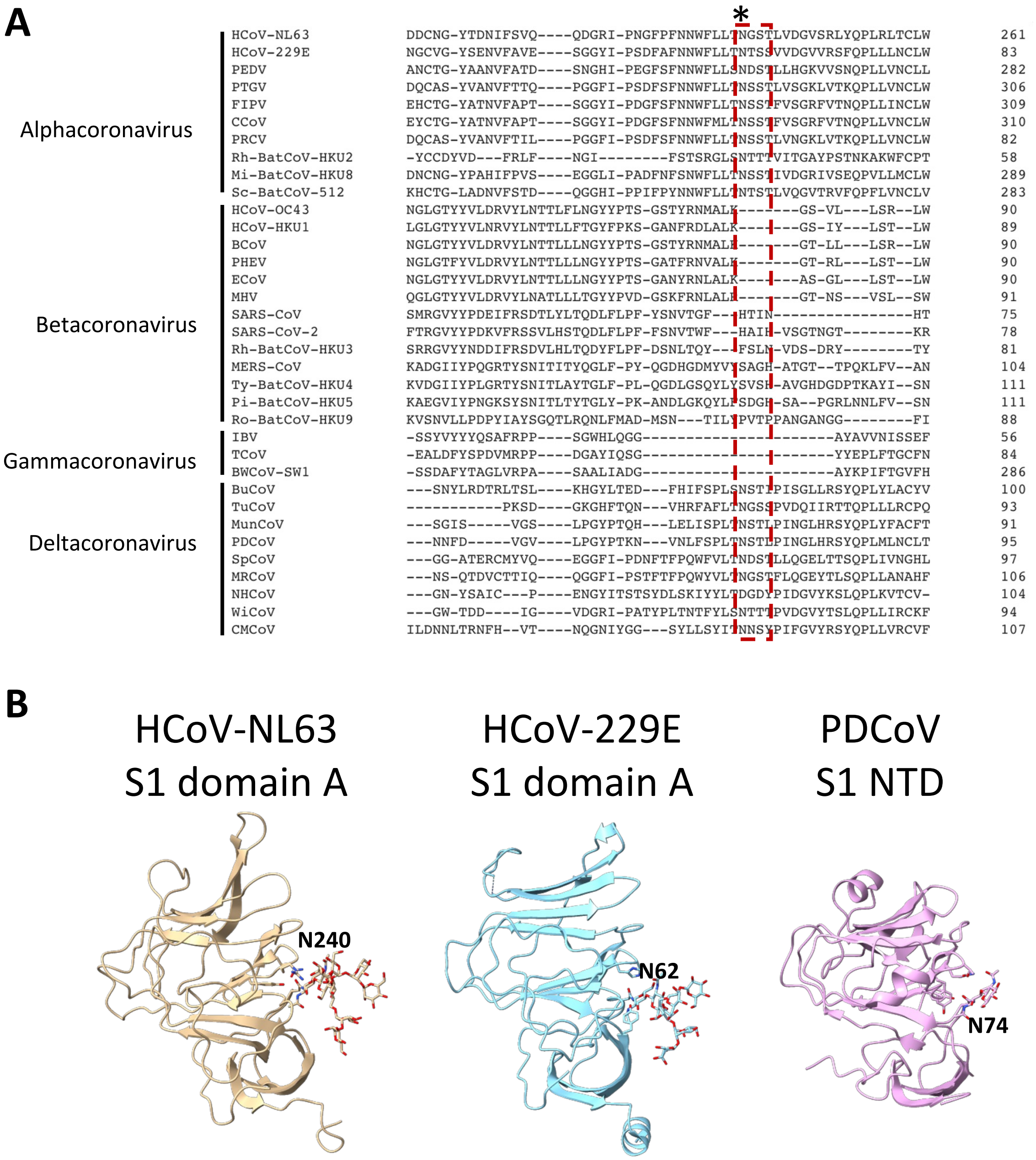

Supplementary Figure 5. One N-linked glycan is conserved among alpha- and delta-coronaviruses (related to Figure 2). (A) Spike sequence alignment among coronaviruses across different genera. N-linked glycosylation sequon at residue 240 of HCoV-NL63 S and aligned coronaviruses is boxed in red. (B) Structures of domain A of HCoV-NL63 and HCoV-229E (PDB:7CYC) and the N-terminal domain of porcine deltacoronavirus (PDCoV)(PDB:6B7N). The conserved N-linked glycans are displayed.

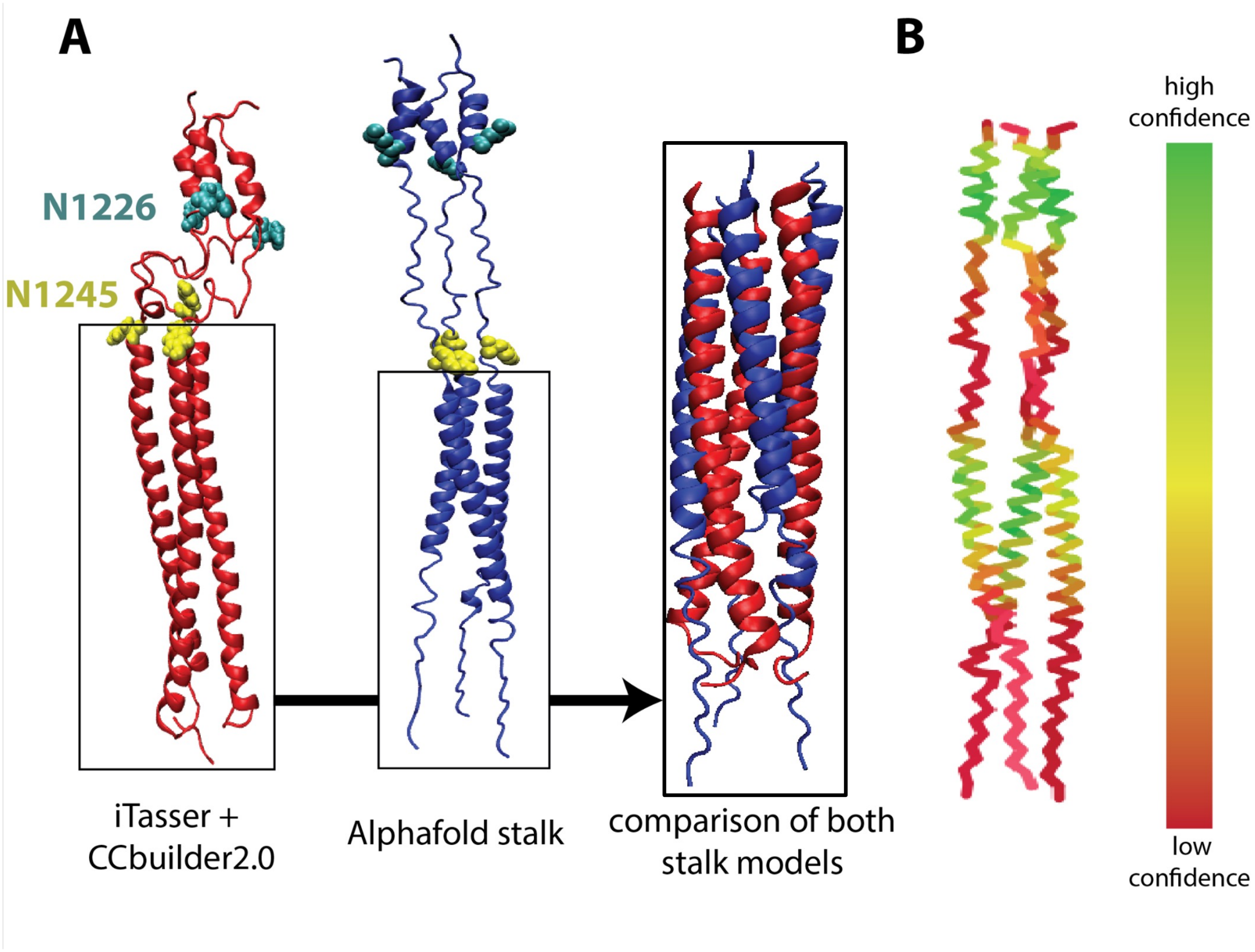

Supplementary Figure 6. Simulated stalk comparison to predicted alphafold structure (related to the integrative modeling section and Figure 3). (**A)** The simulated stalk by the iTasser + CCbuilder2.0 model (left panel) was compared to the stalk produced by alpha fold (middle panel). The upper and lower boundaries of the unstructured region, 1226 (cyan) and 1245 (yellow) respectively, are signified by colored side chains (van der waals representation). The lower stalk regions are superimposed (right panel) with the simulated stalk colored in red and the alphafold output colored in blue. (**B)** The alphafold predicted stalk colored by pLDDT, a measure of structure prediction confidence.

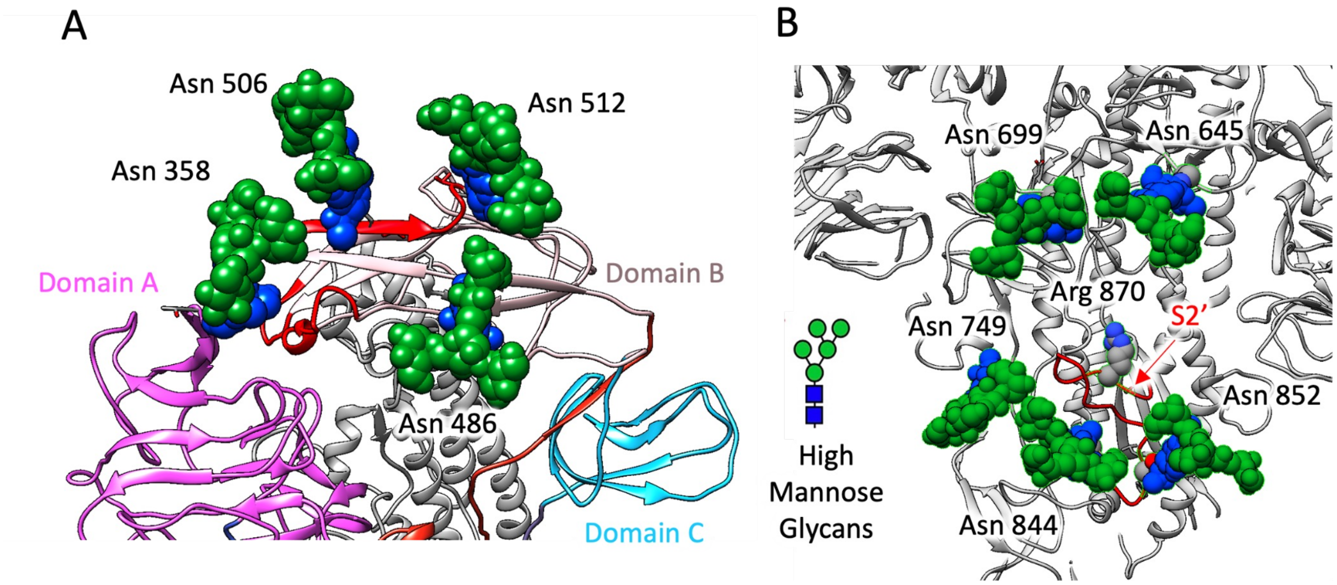

Supplementary Figure 7 (related to Figure 3). (A) Glycans (colored in green and blue) attached to Asn 358, 486, 506 and 512 capping the receptor binding motifs (colored in red) on Domain B. **(B)** A cluster of N-linked glycan gates the S2' cleavage site. The cleavage activation loop upstream of S2' cleavage site is colored in red.

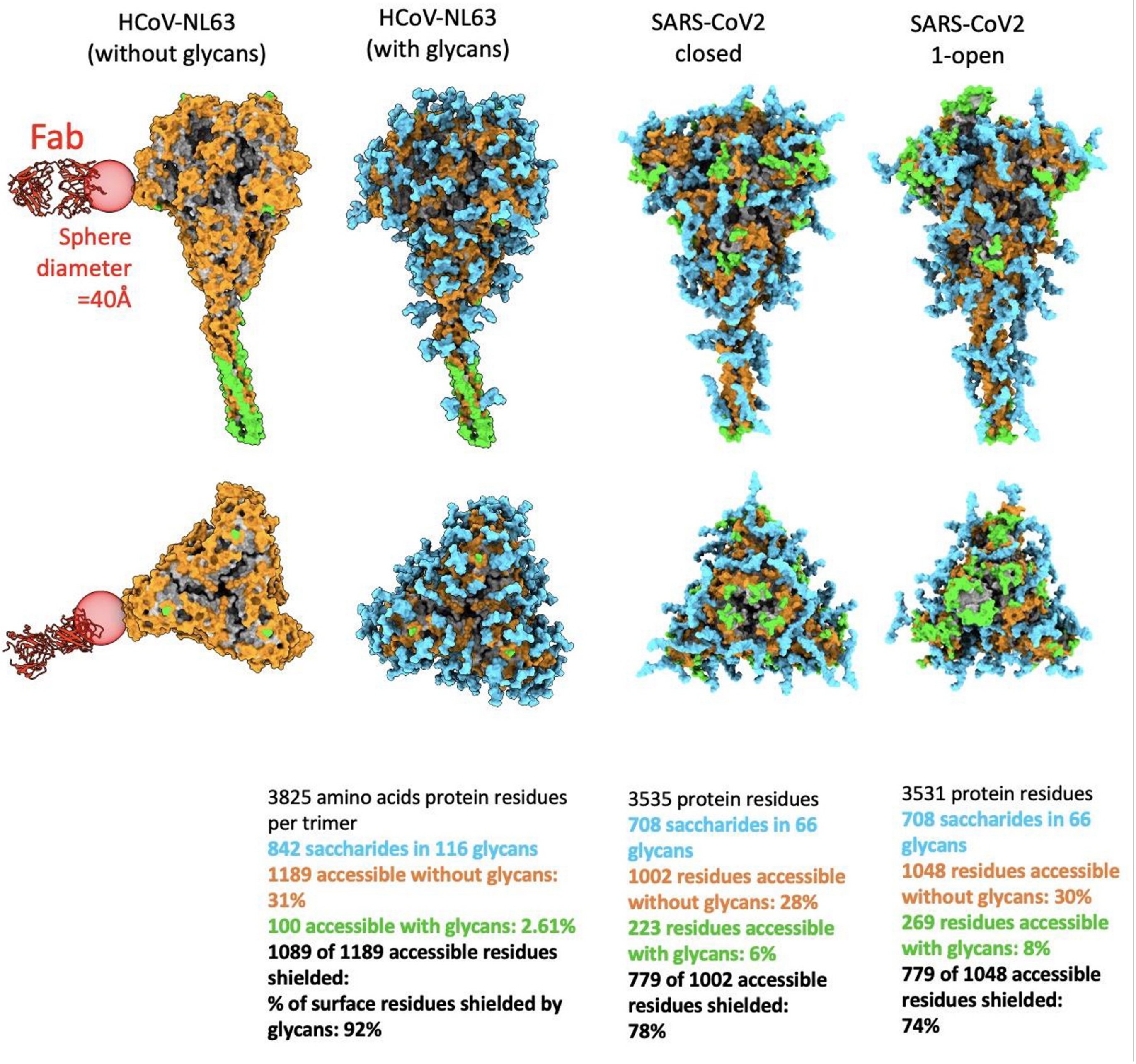

Supplementary Figure 8. (related to Figure 3) Display of the spikes of HCoV-NL63, SARS-CoV-2 in closed and open states to illustrate the accessible surface areas to complementarity-determining regions (CDRs) of a Fab modeled as a sphere of 40Å diameter. Annotations of different colors are explained in each panel.

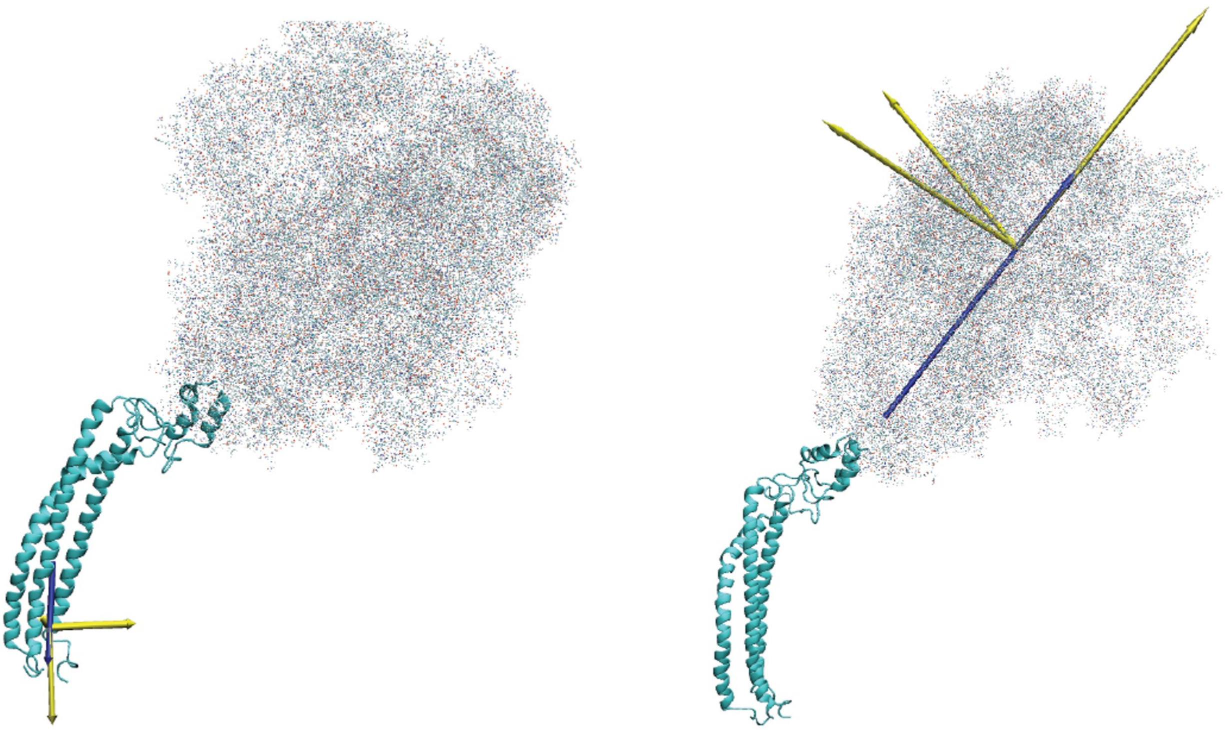

Supplementary Figure 9. Vectors for bending angle measurements (related to Figure 4). Bending in HCoV-NL63 simulations was calculated by finding the arc cosine of two vectors (Blue arrows): one running through the center of the crown and one aligned with the base of the stalk. These vectors were compared to principal axes calculated over the same region (Gold arrows).

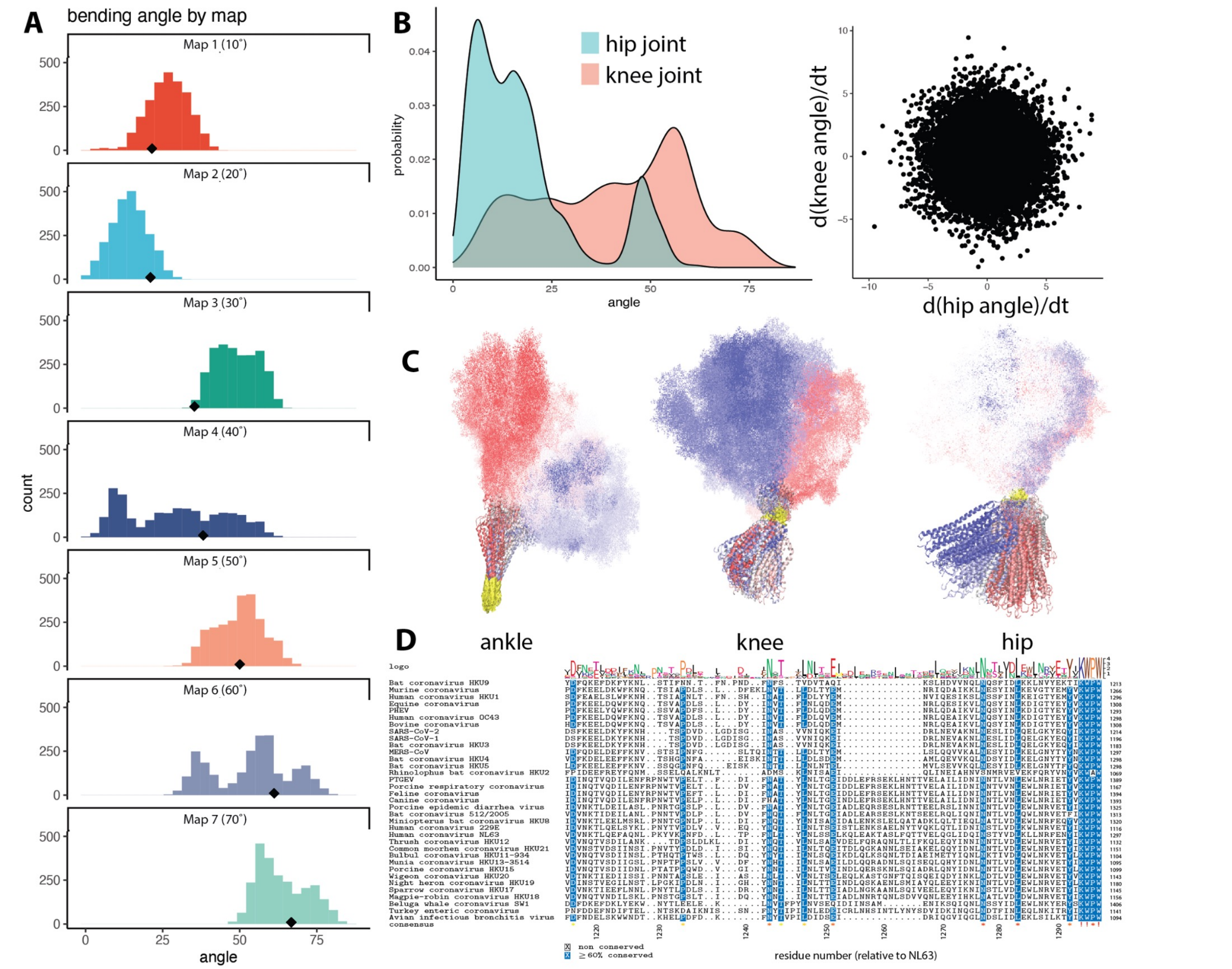

Supplementary Figure 10. Individual map bending angles and analysis of alternative bending hinges (related to Figure 4). **(A)** The individual bending angle distribution experienced during explicit molecular dynamics simulation for all seven tilt maps (Figure 1G, 4A inset). The diamond indicates the bending angle of the structure after energy minimization and thermalization, prior to explicit equilibrium MD simulation. **(B)** Bending angles were measured individually between the hip joint (near the base of the crown) and the knee joint (the unstructured region). The rate of angle change of the hip joint was plotted against the rate of angle change observed in the knee joint (**scatter plot**). (**C**) An ensemble of molecular structures, derived by concatenating all the simulation trajectories described in panel A, were aligned with respect to the hip, knee, or ankle joints (aligned regions highlighted in yellow, *Turoňová et al. Science 2020*). The color scale indicates a bending transition from tilt of 10˚ (red) to 70˚ (blue). (**D**) The multiple sequence alignments for 35 different coronaviruses. Blue shade regions indicate highly conserved residues. Source data are provided as a Source Data file.

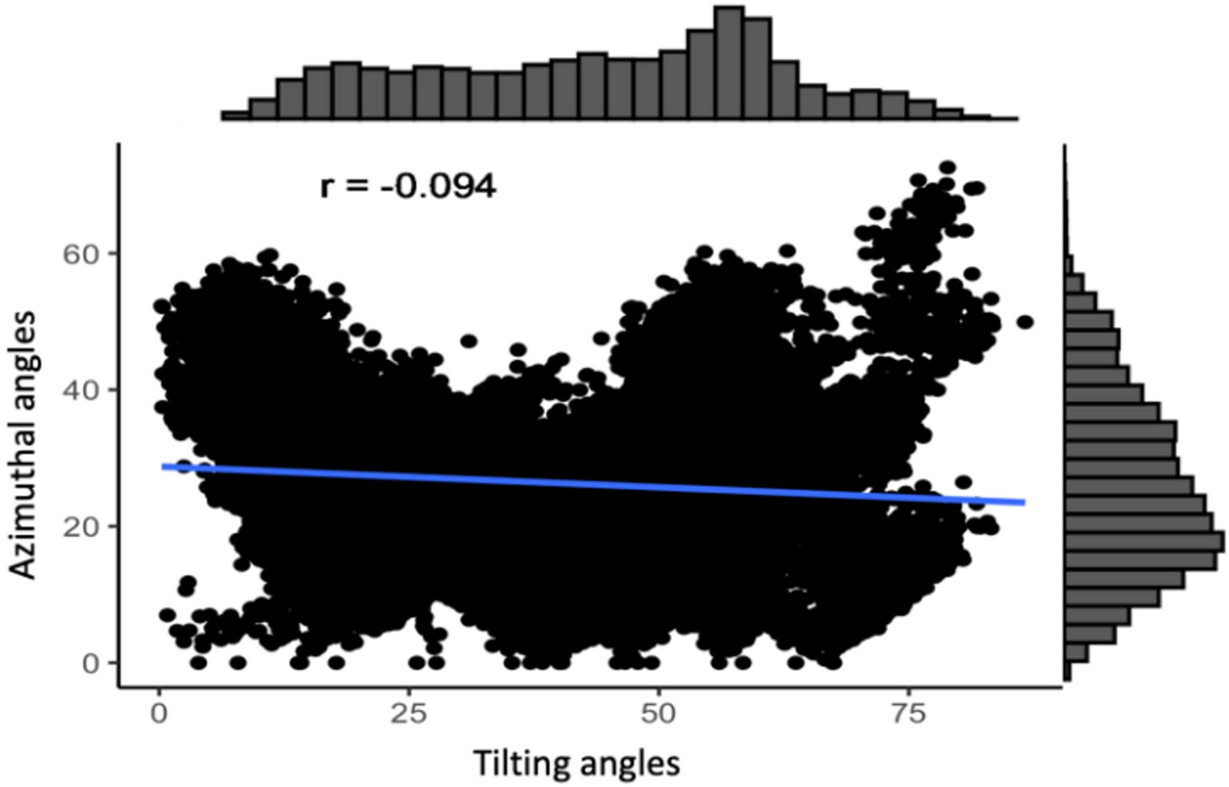

Supplementary Figure 11 (related to Figure 4). Distribution of azimuthal and tilt angles sampled during MD simulations, and a scatter plot showing minimal correlation between the dynamics of these two angles during MD. Source data are provided as a Source Data file.

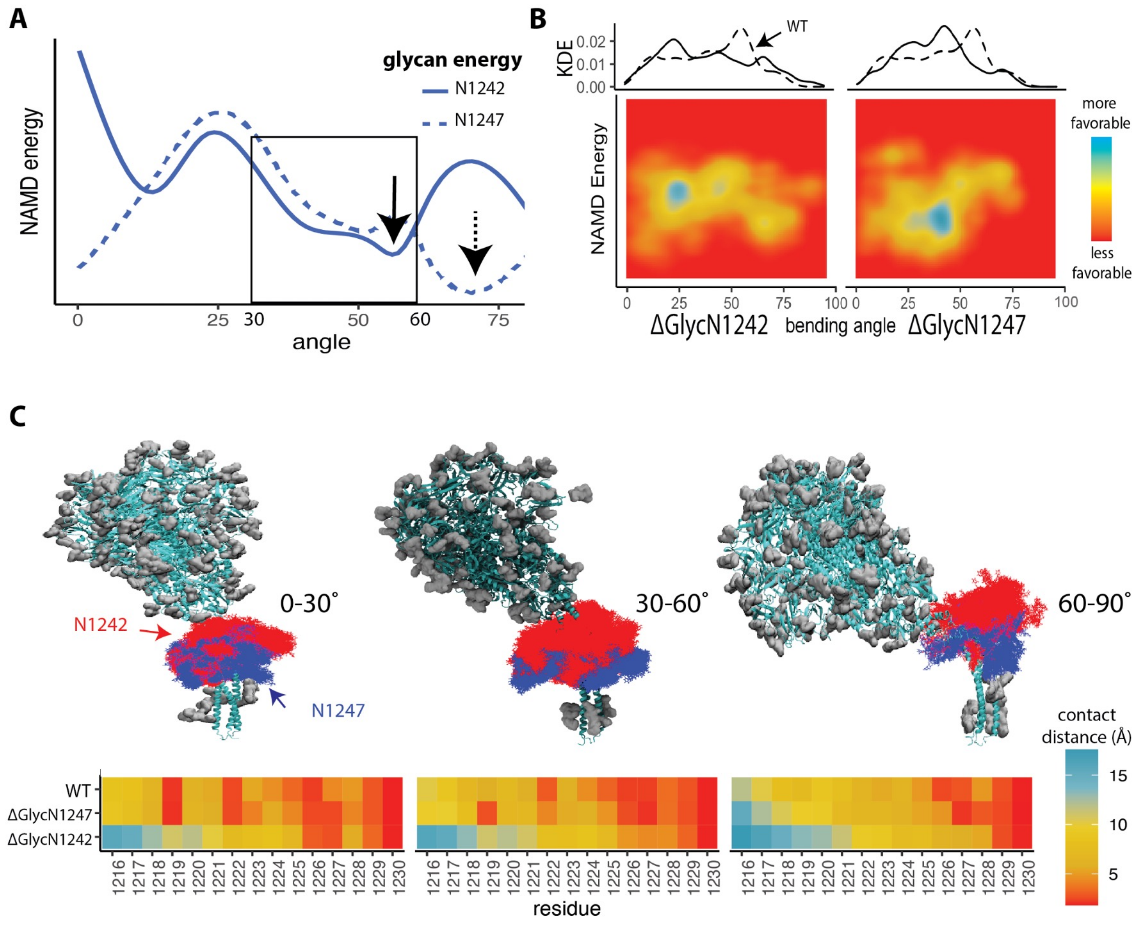

Supplementary Figure 12. Glycan-protein interactions, distances and properties at different bending angles (related to Figure 4). (**A**) Individual hinge glycan energy, with respect to the rest of the system, was measured as a function of bending angle. The arrows indicate the global minima for each glycan (**B**) Contour plots of hinge glycan-protein interaction energy as a function of bending angle for the single hinge glycan deletion (ΔGlycN1242 & ΔGlycN1247) simulations. Blue regions indicate energetically more favorable conformations, while red indicates unfavorable ones. The favorable region shifts from ~25˚ to ~40˚ when comparing ΔGlycN1242 to ΔGlycN1247. The margin distribution of bending angle (measured as kernel density estimate or KDE) is shown as inset on top of each contour. (**C**) Ensemble of N1242 (red) and N1247 (blue) glycans collected across bending angles between 0-30˚, 30-60˚ and 60-90˚ superimposed with protein (in cyan)-glycan (in grey) conformations at representative bending angles within these ranges. Frames from the wild type simulations were saved and structures were sorted into three equally spaced bins. The heatmap depicts the minimum contact distances between hinge glycans and the upper stalk region (1216-1230) for structures falling within the most common bending angles were measured for WT and single glycan deletions. Red regions indicate stalk residues that have a low median contact distance between the hinge glycans and upper stalk region while blue represents the inverse. Source data are provided as a Source Data file.

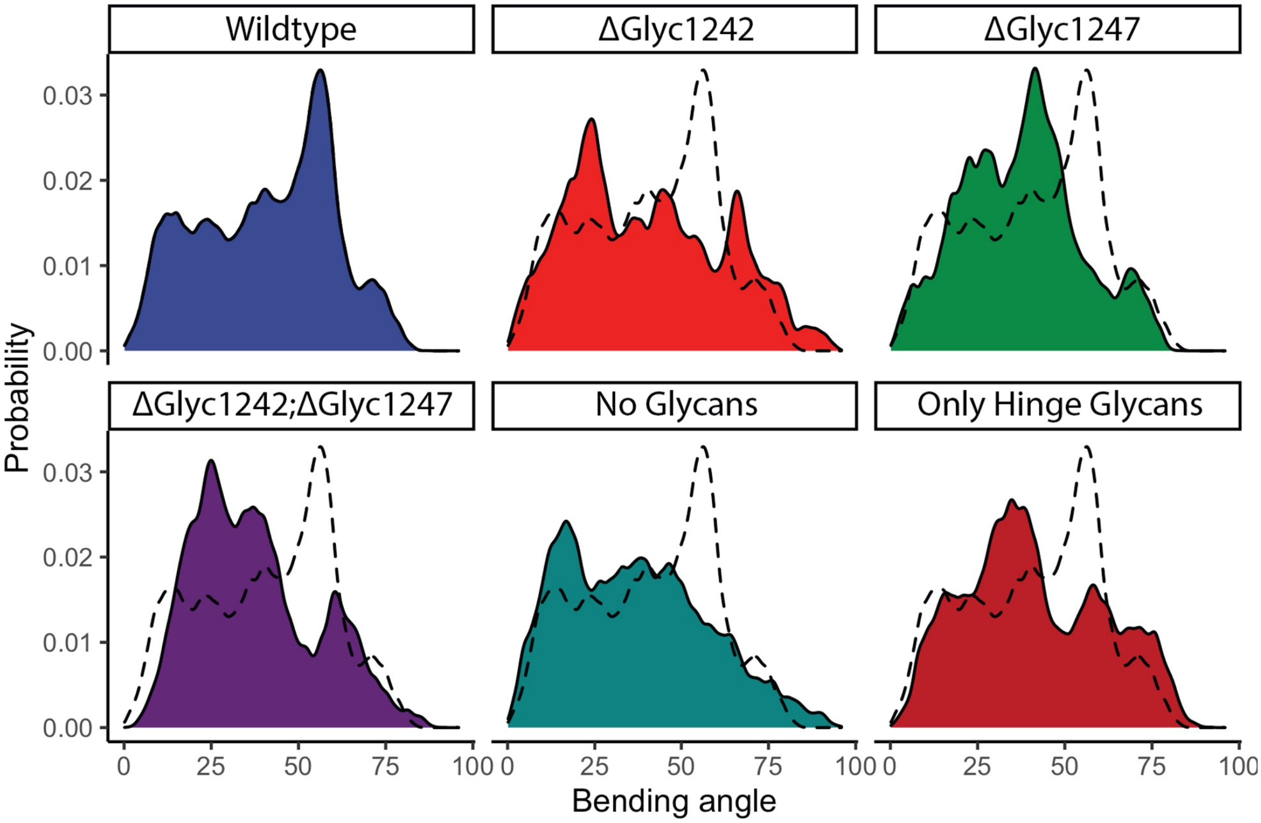

Supplementary Figure 13. Bending angles of HCoV-NL63 following glycan modification (related to Figure 5). Each panel indicates the bending angle distribution observed in MD simulations following hinge glycan modifications. The dashed line depicts the wildtype bending angle distribution. Source data are provided as a Source Data file.

Supplementary Table 1

CryoET data collection and processing parameters

Specimen	HCoV-NL63 virion S trimer
EMDB	EMDB-22889
Data collection and processing	
Magnification	53,000X
Voltage (kV)	200
Energy filter slit width (eV)	20
Detector	K2 Summit
Defocus range (μm)	-2 to -5.5
Pixel size (Å)	2.20
Total electron exposure (e^-/ Å^2)	120
Subtomogram averaging software	EMAN2
Total Number of Particles	18,356
Symmetry imposed	C3
Map resolution (Å) @ FSC 0.143	6.9

Supplementary Table 2

Molecular dynamic simulation details

Explicit solvent									
Glycan configuration		map 1	map 2	map 3	map 4	map 5	map 6	map 7	total (ns)
	replica 1	45	45	51	45	72	47	62	
wild type	replica 2	51	45	49	45	52	55	56	1081
	replica 3	60	39	72	47	52	50	41	
**Implicit solvent**									
Glycan configuration		map 1	map 2	map 3	map 4	map 5	map 6	map 7	total (ns)
	replica 1	100	100	100	95.1	100	100	100	
wild type	replica 2	99.9	100	100	100	92.4	100	100	2085.7
	replica 3	100	100	100	100	98.3	100	100	
	replica 1	97.8	100	100	100	98.7	100	100	
del1242; del1247	replica 2	100	100	100	100	100	100	100	2091.4
	replica 3	100	100	94.9	100	100	100	100	
	replica 1	100	100	100	100	100	100	100	
del1242	replica 2	100	100	100	100	100	100	100	2100
	replica 3	100	100	100	100	100	100	100	
	replica 1	93.4	100	100	100	95.5	100	100	
del1247	replica 2	100	100	100	100	100	100	100	2087.2
	replica 3	100	100	100	100	100	98.3	100	
