# Supplementary material for: Structural insights into the modulation of coronavirus spike tilting and infectivity by hinge glycans
Source: Nat Commun. 2023 Nov 7;14:7175. doi: 10.1038/s41467-023-42836-9 (PMC10630519; doi:10.1038/s41467-023-42836-9)
Supplement: Supplementary file 3 — Description of additional supplementary files [file 41467_2023_42836_MOESM3_ESM.pdf]

Description of Additional Supplementary Files

Supplementary Data 1:
Analysis of glycans associated with HCoV-NL63 spike by Mass Spectrometry.

Supplementary Movie 1: A representative 3D cryogenic electron tomogram of HCoV-NL63 spikes embedded on the virus membrane.

Supplementary Movie 2: Model of protein and glycan in HCoV-NL63 spike crown derived from cryoEM map and glycan mass spec. The model of the HCoV-NL63 spike crown is shown, using a surface representation of the cryoEM densities; each of the three spike proteins has a different color. Models of glycans are shown emerging from these surfaces, displayed using a stick model. CryoEM density of resolved glycans is shown with a yellow surface. The unresolved glycan models are based on data from mass spectroscopy (MS).

Supplementary Movie 3: Animation of Molecular Dynamic Simulations of bending of wild type HCoVNL63 spike (red) vs spike without the hinge glycans (blue) showing differences in the flexibility of the models.
