# Supplementary material for: Structural insights into the modulation of coronavirus spike tilting and infectivity by hinge glycans
Source: Nat Commun. 2023 Nov 7;14:7175. doi: 10.1038/s41467-023-42836-9 (PMC10630519; doi:10.1038/s41467-023-42836-9)
Supplement: Supplementary file 8 — Reporting Summary [file 41467_2023_42836_MOESM8_ESM.pdf]

nature portfolio

Corresponding author(s): Wah Chiu, Abhishek Singharoy, Jing Jin

Last updated by author(s): 09/28/2023

Reporting Summary

Nature Portfolio wishes to improve the reproducibility of the work that we publish. This form provides structure for consistency and transparency in reporting. For further information on Nature Portfolio policies, see our Editorial Policies and the Editorial Policy Checklist.

Statistics

For all statistical analyses, confirm that the following items are present in the figure legend, table legend, main text, or Methods section.

n/a	Confirmed	
☐	☒	The exact sample size (*n*) for each experimental group/condition, given as a discrete number and unit of measurement
☐	☒	A statement on whether measurements were taken from distinct samples or whether the same sample was measured repeatedly
☐	☒	The statistical test(s) used AND whether they are one- or two-sided<br>*Only common tests should be described solely by name; describe more complex techniques in the Methods section.*
☐	☒	A description of all covariates tested
☐	☒	A description of any assumptions or corrections, such as tests of normality and adjustment for multiple comparisons
☐	☒	A full description of the statistical parameters including central tendency (e.g. means) or other basic estimates (e.g. regression coefficient) AND variation (e.g. standard deviation) or associated estimates of uncertainty (e.g. confidence intervals)
☐	☒	For null hypothesis testing, the test statistic (e.g. *F*, *t*, *r*) with confidence intervals, effect sizes, degrees of freedom and *P* value noted<br>*Give P values as exact values whenever suitable.*
☒	☐	For Bayesian analysis, information on the choice of priors and Markov chain Monte Carlo settings
☒	☐	For hierarchical and complex designs, identification of the appropriate level for tests and full reporting of outcomes
☒	☐	Estimates of effect sizes (e.g. Cohen's *d*, Pearson's *r*), indicating how they were calculated

Our web collection on statistics for biologists contains articles on many of the points above.

Software and code

Policy information about availability of computer code

Data collection

Serial EM software v3.7 (http://bio3d.colorado.edu/SerialEM/) and Tomography software 4.0 (ThermoFisher Scientific) were used to collect electron cryotomography tilt series data.

Molecular dynamics simulations were performed NAMD 2.14 and NAMD 3.0 (https://www.ks.uiuc.edu/Research/namd/)

Data analysis

For cryoET data processing, MotionCor2 software v1.3.0 (https://emcore.ucsf.edu/ucsf-software) was used to align frames of each tilt image. Automated tilt series alignment and reconstruction were performed in IMOD software v4.7 (http://bio3d.colorado.edu/imod/) and EMAN2 software v2.3 (https://blake.bcm.edu/emanwiki/EMAN2).

Subtomogram averaging was performed in EMAN2 and subvolume analysis was performed in the EMAN2 python environment. Visualization, figure generation and model docking were performed in UCSF Chimera v1.16 and UCSF ChimeraX v1.3.

The MS spectra of the deglycosylated HCoV-NL63 spike were analyzed using SEQUEST (Proteome Discoverer 2.5, Thermo Fisher Scientific) and pGlyco v3.076 was used for glycopeptide database searches.

Statistical analysis was performed with Prism 9 v9.4.1 (GraghPad Software, LLC.).

Saccharaide molecules were generated with phenix.elbow (Phenix v1.19). Modeling of saccharides into full glycans was performed with SegMod in Segger v2.9.4, a plugin for UCSF Chimera; code and instructions available on GitHub https://github.com/gregdp/segger.

Refinement was performed with phenix.real_space_refine (Phenix v1.19).

Model building was done using CCbuilder2.0 (http://coiledcoils.chm.bris.ac.uk/ccbuilder2/builder), iTASSER (https://zhanggroup.org/I-TASSER/) , and Alphafold2 (https://github.com/deepmind/alphafold).

All molecular visualizations and analysis were performed using the VMD 1.9.3 analysis tool (https://www.ks.uiuc.edu/Research/vmd/).

For manuscripts utilizing custom algorithms or software that are central to the research but not yet described in published literature, software must be made available to editors and reviewers. We strongly encourage code deposition in a community repository (e.g. GitHub). See the Nature Portfolio guidelines for submitting code & software for further information.

Data

Policy information about availability of data

All manuscripts must include a data availability statement. This statement should provide the following information, where applicable:

- Accession codes, unique identifiers, or web links for publicly available datasets
- A description of any restrictions on data availability
- For clinical datasets or third party data, please ensure that the statement adheres to our policy

CryoEM maps reported in this study are deposited in the Electron Microscopy Data Bank (EMDB) under the following accession codes: (EMDB-29395); Atomic model of spike protein and glycans is deposited to wwProtein Data Bank (PDB:8FR7). The mass spectrometry data have been deposited to the ProteomeXchange Consortium via the PRIDE partner repository with the dataset identifier PXD039247.

Human research participants

Policy information about studies involving human research participants and Sex and Gender in Research.

Reporting on sex and gender	not applicable
Population characteristics	not applicable
Recruitment	not applicable
Ethics oversight	not applicable

Note that full information on the approval of the study protocol must also be provided in the manuscript.

Field-specific reporting

Please select the one below that is the best fit for your research. If you are not sure, read the appropriate sections before making your selection.

☒ Life sciences ☐ Behavioural & social sciences ☐ Ecological, evolutionary & environmental sciences

For a reference copy of the document with all sections, see nature.com/documents/nr-reporting-summary-flat.pdf

Life sciences study design

All studies must disclose on these points even when the disclosure is negative.

Sample size	The sample size was determined by the available electron microscopy time and the amount of virus sample. The sample size was sufficient to obtain a structure at the resolution stated in the manuscript, to obtain a biological conclusion. The mass spectrometry glycoform analyses were carried out using single samples, which could be produced reliably using the protocols defined in the Methods section.
Data exclusions	For cryoET data, individual tilt images or tilt series were omitted if they contained artifacts such as incorrectable drift or locations without any specimen.
Replication	The EM data here was collected from 161 reconstructed tomograms containing approximately 15,000 individual spike particles. CryoEM data was collected in 10+ independent sessions over 10+ individual cryoEM grids following independently reproducible replications of the experiments.<br><br>Eight aliquots of HCoV-NL63 S protein were subjected to glycopeptide analysis and six aliquots were subjected to peptide analysis followed by reduction.<br><br>The pseudovirus infection experiment was repeated three times and in each experiment samples were triplicated.<br><br>All NAMD simulations were performed in triplicate.<br><br>All attempts at replication were successful.

Randomization	For cryoET data processing, subvolume particles were randomly assigned into two independent datasets and each dataset was refined independently to satisfy the gold-standard FSC criterion for resolution determination.<br>Randomization is not applicable to other experiments in this work.
Blinding	Subvolume particles were picked from tomograms of purified HCoV-NL63 virions based on their features and then subjected to subvolume averaging and classification with EMAN2 software based on their 3D features. Blinding in investigation is not relevant to our study.

Reporting for specific materials, systems and methods

We require information from authors about some types of materials, experimental systems and methods used in many studies. Here, indicate whether each material, system or method listed is relevant to your study. If you are not sure if a list item applies to your research, read the appropriate section before selecting a response.

Materials & experimental systems

n/a	Involved in the study
☒	☐ Antibodies
☐	☒ Eukaryotic cell lines
☒	☐ Palaeontology and archaeology
☒	☐ Animals and other organisms
☒	☐ Clinical data
☒	☐ Dual use research of concern

Methods

n/a	Involved in the study
☒	☐ ChIP-seq
☒	☐ Flow cytometry
☒	☐ MRI-based neuroimaging

Eukaryotic cell lines

Policy information about cell lines and Sex and Gender in Research

Cell line source(s)	Hamster fibroblast cell line BHK21 (Cat. #CCL-10), African green monkey kidney epithelial cell line MA104 (ATCC CRL-2378.1) and adenovirus 5 DNA transformed human kidney epithelial cell line 293 c18 (Cat. #CRL-10852) were purchased from American Type Culture Collection (ATCC). 293-ACE2-TMPRSS2 cell line was established by transducing 293 c18 cells with lentivirus expressing ACE2 and TMPRSS2 followed by selection under blastidicin and puromycin.
Authentication	All the cell line ordered from ATCC were authenticated using ATCC Cell Line Authentication Service with STR profiling. The 293-ACE2-TMPRSS2 stable cell line established in the lab was confirmed for ACE2 and TMPRSS2 over-expression by Western Blot analysis of cell lysate.
Mycoplasma contamination	All the cell line used in the study were confirmed mycoplasma negative with Myco-sniff-valid mycoplasma PCR detection kit (MP bio, SKU093050301).
Commonly misidentified lines<br>(See ICLAC register)	no commonly misidentified cell lines were used in the study

March 2021
